# Risk factors for precancerous cervical lesion among women screened for cervical cancer in south Ethiopia: Unmatched case-control study

**DOI:** 10.1371/journal.pone.0254663

**Published:** 2021-07-15

**Authors:** Tesfalidet Beyene, Mohammed Akibu, Henok Bekele, Wengelawit Seyoum

**Affiliations:** 1 College of Medical and Health Sciences, Wollega University, Nekemte, Oromia, Ethiopia; 2 Postdoctoral Research Fellow, University of Newcastle, Callaghan, Australia; 3 Department of Midwifery, Institute of Medicine and Health Sciences, Debre Berhan University, Debre Birhan, Ethiopia; 4 Pharma College, Hawassa, Ethiopia; Mbarara University of Science and Technology, UGANDA

## Abstract

**Background:**

Nearly 90% of deaths from cervical cancer occur in a low resource setting. In Ethiopia, the magnitude of precancerous cervical lesions ranges from 7% to 28%. Precancerous cervical lesions may progress to cervical cancer. Early screening and treatment of precancerous cervical lesions is a cost-effective way to avert the growth of cervical cancer. However, there has been limited research on risk factors for precancerous cervical lesions in Ethiopia. Therefore, this study aimed to identify risk factors for precancerous cervical lesions among women screened for cervical cancer in south Ethiopia.

**Method:**

A facility-based unmatched case-control study was carried out in five health facilities in south Ethiopia between 8 May to 28 September 2018. Interviewer administered questionnaires were used to collect data from 98 cases and 197 controls. Multivariate logistic regression was employed to identify determinants of precancerous cervical lesions.

**Results:**

Women aged 30–39 years (AOR = 2.51, 95% CI: 1.03–6.08), monthly income ≤66 (AOR = 3.51, 95% CI: 1.77–6.97), initiation of first sexual intercourse at age less than or equal to 20 (AOR = 2.39, 95% CI: 1.14–5.47), having more than one lifetime sexual partner (AOR = 4.70, 95% CI: 2.02–10.95), having a partner/ husband with more than one lifetime sexual partner (AOR = 2.98, 95% CI: 1.35–6.65) had higher odds of precancerous cervical lesions.

**Conclusion and recommendation:**

Strategies to prevent precancerous cervical lesions should focus on modification of lifestyle and sexual behaviour. The findings of this study highlight several implications for policymakers: targeting older women for cervical cancer screening, addressing inequalities and education relating to risky sexual behaviour may reduce precancerous cervical lesions. Furthermore, future longitudinal studies are needed to assess the awareness of women about cervical cancer screening.

## Background

Worldwide, human papillomavirus (HPV) is the most prevalent human viral infections that can infect the skin and mucous membranes [1, 2]. HPV is transmitted via skin-to-skin and sexual contact [2–4]. More than 130 HPV types have been well known and categorized into low or high-risk groups according to their potential for oncogenesis [2, 5]. HPV is one of the most common causes of cervical cancer and precursor lesions [6]. HPV16 and HPV18 are the two most oncogenic types and are responsible for 70% of cervical carcinomas reported globally [7].

Cervical cancer is the fourth most common cancer in women with an estimated 570,000 new cases worldwide in 2018, resulting in 6.6% of all women cancers [8]. Without early treatment and prevention, the annual number of new cervical cancer cases is estimated to rise from 570,000 to 700,000 between 2018 and 2030, while the annual number of deaths is expected to increase from 311,000 to 400,000 [1]. Every minute of every day, a new case of cervical cancer affects a woman [9]. According to the World Health Organization (WHO); ninety per cent of deaths from cervical cancer occur in low resource settings [8]. In low and lower-middle-income countries cervical cancer is the first and second highest cause of cancer deaths, respectively [1]. Sub-Saharan Africa has the most burden of cervical HPV infection in Mozambique (41%) and Guinea (48%) [9, 10]. With early detection and effective treatment, cervical cancer is one of the most avoidable and treatable cancer types. However, the majority of women with cervical cancer in low-income countries are diagnosed and managed at the late stage of the disease and do not have access to lifesaving prevention and treatment options [11].

In low resource settings, detection of precancerous cervical lesions may be accomplished using Visual inspection of the cervix using acetic acid (VIA). Cervical cancer screening with VIA is simple and affordable and can be combined with simple treatment for early cervical lesions [12, 13]. Further, its results are available immediately, which gives the opportunity to treat women with VIA positives the same day where possible [13].

In Ethiopia, cervical cancer ranks as the second leading cause of female cancer after breast cancer in women aged 15 to 44 years [14]. The incidence and mortality from cervical cancer is 26 and 18 per 100,000, respectively [15]. These numbers are likely to be under-reported given the limited access to screening services and low level of awareness of cervical cancer [16]. Studies in Ethiopia have shown that the magnitude of precancerous cervical lesions among women ranges from 7% to 28% [17–20]. This indicates the high burden of the disease in the country. In 2010, Ethiopia implemented a cervical cancer prevention and control program and recommended women be screened for cervical cancer at least every five years following normal results irrespective of HIV status [21]. However, awareness, uptake and acceptance of cervical cancer screening is low [22–25].

The literature has reported risk factors that have been linked to cervical cancer and its precursors [26, 27]. The risk for developing cervical cancer is associated with multiple sexual partners, sexually transmitted infection (STI), and the early age of first intercourse [26, 28, 29]. There has been limited research in Ethiopia on the risk factors of precancerous cervical lesions [26, 30]. In Ethiopia, research has focused on awareness, prevalence, determinants of cervical cancer screening and risk factors of cervical cancer in Human Immunodeficiency Virus (HIV) positive women [22, 23, 30, 31]. To the best of our knowledge, this is the first study to identify determinants of precancerous cervical lesions among women screened for cervical cancer in south Ethiopia. The findings of this study will provide valuable input for health care providers and policymakers in order to prevent precancerous cervical lesions that result in cervical cancer. This study gives important information for health care providers who are working on cancer prevention and treatment by showing the most vulnerable group for a precancerous cervical lesion that might progress to cervical cancer. It assists policymakers to develop early prevention and treatment strategy by identifying those who are valuable to the disease. Further, knowledge on risk factors for precancerous lesion will help women to seek early treatment for cervical cancer and to avoid risk factors that might expose them to the disease. Therefore, we aimed to conduct a study to address the risk factors for precancerous cervical lesions among women screened for cervical cancer in south Ethiopia.

## Methods

### Study setting and study participants

This was an unmatched case-control study involving women aged 21–49 years who had undergone screening for precancerous cervical lesions by VIA. The study was conducted between 8 May to 28 September 2018 at five selected health facilities in Southern Nations, Nationalities and Peoples’ Region (south Ethiopia), Ethiopia. The capital city of the region is Hawassa, which is 285 km from Addis Ababa. According to the Central Statistical Agency of Ethiopia (CSA) report, south Ethiopia has a total population of 15 million, of whom 50.3% are women. The selected health facilities were Hawassa University Comprehensive Specialized Hospital, Durame General Hospital, Arba Minch General Hospital, Adare Hospital, and Yirgalem Hospital. The health facilities are integrated to link any eligible and high-risk women to cervical screening service units from the family planning unit, antenatal care unit, gynaecology care unit, and outpatient departments.

All participants who visited the health facilities for family planning, antenatal care, gynaecologic examination, and other health conditions and got screened for cervical cancer were included in this study. The study participants were identified from those screened by VIA. The VIA procedure was performed by trained midwives or nurses working in the hospital. The results of the VIA were classified as negative, positive, or VIA inconclusive, or suspected of having cancer based on a visual examination [32]. VIA positive is defined as the presence of acetowhitish lesion with well-defined margins observed within the vicinity of the transformation zone or if the whole cervix or cervical growth turned white. VIA negative is defined by the absence of acetowhitish lesion observed on the cervix. Women with suspected cancer through VIA screening results were referred for further diagnosis and management. Cryotherapy was applied for those with VIA positive results. Further, women with VIA positive results were offered immediate treatment and counselled to return one year later for follow up screening. Cases were all age-eligible (21–49 years) women who have been screened for cervical cancer and tested positive for VIA and controls were all age-eligible (21–49 years) women who presented to the same health facility for cervical cancer screening but tested negative for VIA. The cases and controls were selected from each health facilities using simple random sampling technique. For each case, two controls were selected until the required sample size was reached.

### Sample size

The sample size was calculated with Open Epi version 3 statistical software, Centres for Disease Control and Prevention, USA [33]. Assuming the proportion of controls (women without precancerous cervical lesion) with lifetime number of sexual partner two and above (30.57%), odds ratio 2.17 [26], power 80%, 95% confidence interval, non-response rate 10% and the ratio of cases to control 1:2, our final sample size was 300 (100 cases and 200 controls).

### Data collection tool and technique

Data were collected using an interviewer-administered questionnaire, which was developed after reviewing the literature [26–30]. The questionnaire encompassed socio-demographic characteristics, reproductive characteristics, lifestyle and sexual behaviours. The socio-demographic factors included were age, residence, religion, marital status, educational status, occupation and household monthly income. The reproductive characteristics include parity, age of the women at menarche, menstrual pattern, contraceptive use, birth interval, history of recurrent abortion and age at first pregnancy. Lifestyle and sexual behaviour characteristic include age at first sexual intercourse, lifetime sexual partner, lifetime sexual partners of the husband, history of STI, history of postcoital bleeding, family history of cervical cancer, history for cervical screening, HIV status, history of smoking, history of chronic disease and vulva washing after every sexual intercourse. The questionnaires were developed in English, translated into regional language (Amharic). To maintain consistency, the questionnaire retranslated back to English by people who are proficient in both languages. The interview was conducted in a private room to create confidence within a secure environment.

### Data quality assurance

A pre-test was performed to check the consistency of the tools. Necessary modification had been made to the tools based on the pre-test results. Data were collected by five experienced midwives from each health facility. Training was given for one day in relation to the objective, the significance of the study, confidentiality of information, respecting the rights of the participants and informed consent. Five supervisors were recruited to oversee the data collection process. All the data were checked for clarity, completeness, and consistency by data collectors, supervisors, and the principal investigator.

### Data management and analysis

Data analysis were performed using SPSS version 21 (IBM SPSS Statistics for Windows, Version 21.0. Armonk, NY: IBM Corp). Descriptive statistics are presented by number and percentage for all variables. Both bivariate and multivariate logistic regression analyses were carried out to identify determinants of precancerous cervical lesions. Bivariate analysis was executed between the dependent variable and each of the independent variables. Crude odds ratio (COR) with 95%CI and p-value were calculated to determine the presence and strength of the association. To avoid residual confounding in multivariate analysis; variables with a p-value less than 0.20 in the bivariate analyses were included in multivariable models [34]. Multivariable logistic regression using an adjusted odds ratio (AOR) was carried out to identify the independent determinant of precancerous cervical lesions. A p-value less than 0.05 were considered significant. Multicollinearity was assessed using variance inflation factors, with a threshold of 10 [35]. Furthermore, the Hosmer-Lemeshow Goodness of Fit tests was applied to check the model adequately fits the data.

### Ethical statement

The study was approved by the Research Ethics Committee of the Pharma College, Hawassa, Ethiopia (Pharm 68/2018). Informed verbal consent was obtained from all study participants before conducting the interview. The ethics committees endorsed the verbal consent procedure since the current study was non-invasive and used only interviewing participants and reviewing their medical records. Health workers were independent witness to observe the verbal consent. Consent was asked after the data collectors shared the information sheet with the study participants. When the participants agreed to participate, the data collectors had marked yes and signed in the consent form. Participation in the study was completely voluntary and refusal to respond to the questions from the study was possible.

## Results

A total of 98 cases and 197 controls were recruited with a response rate of 98.7%. The mean and standard deviation of the participant age was 32.7 (5.3). More than two thirds (67.3%) of cases and more than half (59.9%) of controls were in the age group of 30–39 years. Fifty-four (55.1%) cases and 120 (60.9%) controls were from urban residences. Forty-four (44.9%) of cases and more than half (55.8%) controls reported being followers of the Protestant religion. The majority (91.8%) of cases and the majority (90.4%) of controls were married. More than one quarter (26.5%) of cases and 45(22.8%) of controls had primary (grade 5–8) as their highest educational level. By occupation, one-third of both cases and controls were housewives. Thirty-nine (39.8%) of cases and 48(24.4%) of controls had a monthly income of less than 43 USD. In bivariate analysis variables associated with precancerous cervical lesions (p < 0.2) were age of the participants and monthly income. However, residence, religion, marital status, education, and occupation didn’t appear to be associated with precancerous cervical lesion (p >0.2) (**Table 1**).

**Table 1:** Socio-demographic characteristics of women screened for cervical cancer in south Ethiopia, 2018.

Variables	Case n (%)	Control n (%)	Crude OR (95%CI)	P-value
**Age(years)**				
21–29	13(13.3)	62(31.5)	1	
30–39	66(67.3)	118(59.9)	2.67 (1.37–5.21)	0.003
40–49	19(19.4)	17(8.6)	5.33 (2.20–12.93)	< 0.001
Total	98	197		
**Residence**				
Urban	54(55.1)	120(60.9)	1	
Rural	44(44.9)	77(39.1)	1.27 (0.78–2.07)	0.31
Total	98	197		
**Religion**				
Ethiopian Orthodox	32(32.7)	64(32.5)	1	
Muslim	18(18.4)	19(9.6)	1.89 (0.87–4.10)	0.24
Protestant	44(44.9)	110(55.8)	0.8 (0.46–1.39)	0.43
Catholic	4(4.1)	4(2.0)	2.0 (0.47–8.52)	0.47
Total	98	197		
**Marital status**				
Married	90(91.8)	178(90.4)	3.53 (0.43–29.21)	0.25
Single	1(1.0)	7(3.6)	1	
Widowed/divorced/separated	7(7.14)	12(6.09)	1.15 (0.44–3.03)	0.77
Total	98	197		
**Women’s educational level**				
Can’t read and write	17(17.3)	31(15.7)	1	
Able to read and write but with no schooling	15(15.3)	23(11.7)	1.19 (0.49–2.86)	0.70
Primary 1–4	17(17.3)	29(14.7)	1.07 (0.46–2.48)	0.88
Primary 5–8	26(26.5)	45(22.8)	1.05 (0.49–2.26)	0.89
Secondary school	10(10.2)	34(17.3)	0.54 (0.21–1.35)	0.21
College diploma and above	13(13.3)	35(17.8)	0.68 (0.28–1.64)	0.38
Total	98	197		
**Women’s occupation**				
House wife	33(33.7)	65(33)	1	
Government employee	14(14.3)	35(17.8)	0.79 (0.37–1.66)	0.53
Merchant	26(26.5)	40(20.3)	1.28 (0.67–2.45)	0.45
Farmer	11(11.2)	29(14.7)	0.75 (0.33–1.68)	0.48
Daily laborer	5(5.1)	10(5.1)	0.98 (0.31–3.11)	0.98
Student	6(6.1)	9(4.6)	2.90 (0.78–11.20)	0.22
Other	3(3.1)	9(4.6)	0.66 (0.17–2.59)	0.55
Total	98	197		
**Husband or partner educational status**				
Can’t read and write	9(9.3)	12(6.3)	1	
Able to read and write but with no schooling	11(11.3)	15(7.9)	0.98 (0.30–3.13)	0.97
Primary 1–4	14(14.4)	32(16.8)	0.58 (0.20–1.70)	0.32
Primary 5–8	17(17.5)	34(17.9)	0.67 (0.23–1.89)	0.45
Secondary school	19(19.6)	38(20.0)	0.67 (0.24–1.86)	0.44
College diploma and above	27(27.8)	59(31.1)	0.61 (0.23–1.62)	0.32
Total	97	190		
**Income per month (ETB)**				
< = 42 USD	39(39.8)	48(24.4)	2.64 (1.37–5.21)	0.007
43–66 USD	18(18.4)	43(21.8)	1.36 (0.62–2.98)	0.44
67-139USD	25(25.5)	54(27.4)	1.51 (0.72–3.14)	0.27
>139 USD	16(16.3)	52(26.4)	1	
Total	98	197		

1USD = 28.48

### Reproductive characteristics

Fifty-nine (60.2%) of cases and the majority (90.9%) of controls have one to four children. The mean and standard deviation of age at menarche of the participants was 15.2 ± 1.7. More than two-thirds (68.4%) of cases and nearly three quarter (72.1%) of controls reported having a regular menstrual history. Half (51.0%) of cases and nearly half (46.7%) of controls reported currently using contraceptives. Nearly half of the cases and controls currently reported using an injectable contraceptive. More than half of cases and controls ever used oral contraceptive methods. The birth interval was similar between cases and controls, 70.2% and 70.1% gave birth after two years. Most cases (93.9%) and controls (94.4%) had no history of recurrent abortion. More than half (53.1%) of cases and nearly half (48.7%) controls had their first pregnancy before the age of 21 years. In bivariate analysis, parity and ever use of contraceptive methods were found to be associated with precancerous cervical lesions (p <0.2). Nevertheless, age at menarche, menstrual pattern, current use of any form of contraceptive methods, type of contraceptive, birth interval, history of recurrent abortion and age at first pregnancy were not associated with precancerous cervical lesion (p>0.2) (**Table 2**).

**Table 2:** Reproductive characteristics of women screened for cervical cancer in south Ethiopia, 2018.

Variables	Case n (%)	Control n (%)	COR (CI 95%)	p-value
**Parity**				
No	4(4.1)	6(3.0)	1	
1–4	59(60.2)	179(90.9)	0.49 (0.14–1.81)	0.29
> = 5	35(35.7)	12(6.1)	4.38 (1.05–18.19)	0.04
Total	98	197		
**Age of menarche**				
< = 12	6(6.1)	11(5.6)	1	
13–15	50(51.0)	96(48.7)	0.95 (0.33–2.73)	0.93
>15	42(42.9)	90(45.7)	0.85 (0.30–2.47)	0.77
Total	98	197		
**Menstrual pattern**				
Regular	67(68.4)	142(72.1)	1	
Sometimes irregular	20(20.4)	43(21.8)	0.98 (0.54–1.80)	0.96
Always irregular	11(11.2)	12(6.1)	1.94 (0.81–3.63)	0.23
Total	98	197		
**Currently using any form of contraceptive**				
Yes	50(51.0)	92(46.7)	1.19 (0.73–1.93)	0.48
No	48(49.0)	105(53.3)	1	
Total	98	197		
**Type of contraceptive**				
Oral contraceptive	14(28.0)	28(30.4)	1	
Injectable	24(48.0)	46(50.0)	1.04 (0.46–2.34)	0.92
Implants	9(18.0)	13(14.1)	1.38 (0.478–4.01)	0.55
IUCD	3(6.0)	5(5.4)	1.2 (0.25–5.76)	0.82
Total	50	92		
**Ever use of oral contraceptive**				
Yes	58(59.2)	101(51.3)	1.38 (0.84–2.25)	0.20
No	40(40.8)	96(48.7)	1	
Total	98	197		
**Birth interval**				
<24 months	28 (29.8)	56(29.9)	0.99 (0.58–1.70)	0.98
> = 24 months	66(70.2)	131(70.1)	1	
Total	94	187		
**History of recurrent abortion**				
Yes	6(6.1)	11(5.6)	1.10 (0.39–3.07)	0.85
No	92(93.9)	186(94.4)	1	
Total	98	197		
**Age at first pregnancy**				
< = 20	52(53.1)	96(48.7)	1.19 (0.73–1.93)	0.48
>20	46(46.9)	101(51.3)	1	
Total	98	197		

### Lifestyle and sexual behaviours

Eighty-four (85.7%) of cases and 149 (75.6%) of controls reported first sexual intercourse after the age of 20 years. One third (33.7%) of cases and 16 (8.1%) of controls reported having multiple sexual partners. Thirty-six (36.7%) of cases and 37 (18.8%) of controls reported that their husbands or partners had two or more sexual partners. The majority of cases and controls reported no history of sexually transmitted diseases (STD). Seventeen (17.3%) of cases and 17 (8.6%) controls reported that their husband or partner had a history of STD. Most of the cases and controls reported no family history of cervical cancer. Nearly one-sixth of the cases reported a history of post-coital bleeding. Twenty-one (21.4%) of cases reported having ever been screened for cervical cancer. The prevalence of HIV was higher (13.3%) in the cases than in the controls. The majority of cases and controls reported no history of smoking. Nearly three quarters (73.5%) of cases reported that they wash their vulva after sexual intercourse. A statistically significant association was seen between age at first pregnancy, lifetime sexual partner, number of sexual partners of the husband, history of STI, ever history of STI in a sexual partner, history of post-coital bleeding, family history of cervical cancer and HIV status and precancerous cervical lesions (p <0.2). However, there was no statistical association between previous cervical cancer screening, history of smoking, history of chronic disease, and vulva washing after every sexual intercourse and precancerous cervical lesion (p >0.2) (**Table 3**).

**Table 3:** Lifestyle and sexual behaviour characteristics of women screened for cervical cancer in south Ethiopia, 2018.

Variables	Case n (%)	Control n (%)	Crude OR (95%CI)	p-value
**Age at first sexual intercourse**				
< = 20	14(14.3)	48(24.4)	1.93 (1.01–3.71)	<0.001
>20	84(85.7)	149(75.6)		
Total	98	197		
**Life time number of sexual partner**				
No or One	65(66.3)	181 (91.9)	1	
Two or above	33(33.7)	16(8.1)	5.74 (2.97–11.12)	< 0.001
Total	98	197		
**Lifetime sexual partners of the husband**				
No or one	62(63.3)	160(81.2)	1	
Two or above	36(36.7)	37(18.8)	2.51 (1.46–4.33)	< 0.001
Total	98	197		
**History of STI**				
Yes	28(28.6)	23(11.7)	3.03 (1.63–5.61)	< 0.001
No	70(71.4)	174(88.3)	1	
Total	98	197		
**Partner history of STI**				
Yes	17(17.3)	17(8.6)	2.22 (1.08–4.57)	0.030
No	81(82.7)	180(91.4)	1	
Total	98	197		
**History of post-coital bleeding**				
Yes	15(15.3)	13(6.6)	2.56 (1.17–5.62)	0.02
No	83(84.7)	184(93.4)	1	
Total	98	197		
**Family history of cervical cancer**				
Yes	10(10.2)	10(5.1)	2.12 (0.85–5.29)	0.100
No	88(89.8)	187(94.9)	1	
Total	198	197		
**Previously screened for cervical cancer**				
Yes	21(21.4)	35(17.8)	1.26 (0.69–2.31)	0.45
No	77(78.6)	162(82.2)	1	
Total	98	197		
**HIV status**				
Reactive	13(13.3)	7(3.6)	4.15 (1.60–10.78)	0.003
Non-reactive	85(86.7)	190(96.4)		
Total	98	197		
**Ever history of smoking**				
Yes	6(6.1)	7(3.6)	1.77 (0.58–5.42)	0.32
No	92(93.9)	190(96.4)	1	
Total	98	197		
**History of chronic disease**				
Yes	6(6.1)	10(5.1)	1.21 (0.43–3.46)	0.71
No	92(93.9)	187(94.9)	1	
Total	98	197		
**Vulva washing after every sexual intercourse**				
Yes	72(73.5)	135(68.5)	1.27 (0.74–2.18)	0.38
No	26(26.5)	62(31.5)	1	
Total	98	197		

Those respondents in the age of 30–39 years had a higher likelihood of a precancerous cervical lesion compared to those who were in the age group 21–29 and 40–49 years (AOR = 2.51, 95% CI: 1.03–6.08). Respondents having monthly household income ≤66 USD had higher odds to develop a precancerous cervical lesion than those whose monthly household income was more than 66 USD (AOR = 3.51, 95% CI: 1.77–6.97). Respondents who started their first sexual intercourse at age less than or equal to 20 years had higher odds to have a precancerous cervical lesion compared to those who started their first sexual intercourse at an age greater than 20 years (AOR = 2.39, 95% CI: 1.14–5.47). Respondents who had two or more lifetime sexual partners had higher odds to have a precancerous cervical lesion than those who had no and one lifetime sexual partner (AOR = 4.70, CI: 2.02–10.95). Having a partner or husband with two or more other lifetime sexual partners was associated with higher odds to have precancerous cervical lesion (AOR = 2.98 CI: 1.35–6.65). In multivariate analysis, parity, history of STI, ever history of STI in a sexual partner, ever use of oral contraceptive, family history of cervical cancer, history of post-coital bleeding and HIV were not associated with a precancerous cervical lesion (**Table 4**).

**Table 4:** A multivariate logistic regression on determinants of precancerous cervical lesion in south Ethiopia, 2018.

Variables	Case n (%)	Control n (%)	COR (CI 95%)	AOR (CI 95%)
**Age(years)**				
21–29	13(13.3)	62(31.5)	1	1
30–39	66(67.3)	118(59.9)	2.67 (1.37–5.21)	**2.51(1.03–6.08)** *
40–49	19(19.4)	17(8.6)	5.33 (2.20–12.93)	1.92(0.57–6.50)
Total	98	197		
**Parity**				
No	4(4.1)	6(3.0)		1
1–4	59(60.2)	179(90.9)	0.49 (0.14–1.81)	0.16(0.28–1.01)
> = 5	35(35.7)	12(6.1)	4.38 (1.05–18.19)	1.61(0.23–11.48)
Total	98	197		
**Income per month**				
< = 66 USD	39(39.8)	48(24.4)	2.64 (1.37–5.21)	**3.51(1.77–6.97)**
67–139 USD	25(25.5)	54(27.4)	1.51 (0.72–3.14)	2.03(0.79–5.25)
>139 USD	16(16.3)	52(26.4)	1	1
Total	98	197		
**Age at first sexual intercourse (years)**				
< = 20	14(14.3)	48(24.4)	1.93(1.01–3.71)	2.39(1.14–5.47)*
>20	84(85.7)	149(75.6)	1	1
Total	98	197		
**Lifetime sexual partners**				
No or one	65(66.3)	181 (91.9)	1	**1**
Two or above	33(33.7)	16(8.1)	5.74(2.97–11.12)	4.70(2.02–10.95)*
**Number of sexual partners of the husband**				
No or one	62(63.3)	160(81.2)		**1**
Two or above	36(36.7)	37(18.8)	2.51(1.46–4.33)	2.98(1.35–6.65)*
Total	98	197		
**History of STI**				
Yes	28(28.6)	23(11.7)	3.03(1.63–5.61)	2.15(0.57–8.05)
No	70(71.4)	174(88.3)	1	1
Total	98	197		
**Ever history of STD in sexual partner**				
Yes	17(17.3)	17(8.6)	2.22(1.08–4.57)	1.47(0.33–6.56)
No	81(82.7)	180(91.4)	1	
Total	98	197		
**Ever Use of oral contraception**				
Yes	58(59.2)	101(51.3)	1.38(0.84–2.25)	1.07(0.57–1.99)
No	40(40.8)	96(48.7)	1	1
Total	98	197		
**Family history of cervical cancer**				
Yes	10(10.2)	10(5.1)	2.12(0.85–5.29)	2.29(0.73–7.20)
No	88(89.8)	187(94.9)	1	1
Total	98	197		
**History of post-coital bleeding**				
Yes	15(15.3)	13(6.6)	2.56(1.17–5.62)	1.89(0.67–5.35)
No	83(84.7)	184(93.4)	1	1
**HIV status**				
Reactive	13(13.3)	7(3.6)	4.15(1.60–10.78)	3.32(0.96–10.90)
Non-reactive	85(86.7)	190(96.4)	1	1
Total	98	197		

*statistically significant (p < 0.05)

## Discussion

Cancer is progressively a major public health burden in both high income and low-income countries [36]. Globally, the high mortality rate from cervical cancer could be reduced through a comprehensive approach such as identifying the risk factors, prevention and early detection, effective screening and treatment programs [37]. Identifying the risk factors of precancerous cervical lesions is important for policymakers to develop strategies in the prevention of cervical cancer. Thus, this study aimed to identify the risk factors for precancerous cervical lesions in south Ethiopia.

Studies indicated that older women had a higher likelihood of having precancerous cervical lesions than younger women [26, 38]. The present study showed that respondents who were aged 30–39 years had higher odds to have precancerous cervical lesion compared to others. A study done on age-specific incidence of cervical cancer found that cervical cancer increases rapidly with age and usually reached peak at age 40 years [39]. However, there are studies done elsewhere in Ethiopia which reported age had no association with precancerous cervical lesions [30, 40]. The difference may be due to a difference in study participants; the present study included women who were in their reproductive age groups, but the latter studies include only HIV positive women.

Evidence from low and middle-income countries has shown that low individual socioeconomic status influences health outcomes through a lack of awareness about the health impact of lifestyle risk factors and reduced access to healthcare for financial reasons [41–43]. Our study showed that women who reported monthly household income less than or equal to 66 USD had higher odds to have a precancerous cervical lesion compared to those who reported an income of more than 66 USD. A meta-analysis done on the risk of cervical cancer indicated that the risk of having cervical cancer is highest among women who had low income [44, 45]. One possible explanation for this may be that women with low income may not get access to health care services because of financial hardship. Furthermore, financial hardship may influence the health of women by limiting access to information about the health impact of lifestyle risk factors or access to routine screening. This study highlights the importance of reducing inequalities to prevent and enable early detection of precancerous cervical lesions.

Our analysis showed that early initiation of sexual intercourse increases the odds of precancerous cervical lesion which is consistent with studies done elsewhere and in Ethiopia [27–29, 46, 47]. The association between early first sexual intercourse and cervical cancer can be explained by the fact that a biological predisposition of the immature cervix during adolescence might be more susceptible to persistent HPV infections and therefore have a greater risk of cervical cancer development [48, 49]. Our finding was inconsistent compared to the study done in Addis Ababa which indicated that early sexual intercourse was not associated with precancerous cervical lesion [26]. The reason for this difference might be the age classification of the two studies; the present study classified the age for sexual initiation as less than 20 years, while the study in Addis Ababa considered as less than 18 years of age. Further, the study in Addis Ababa included only women from urban areas where early initiation of sexual intercourse was more common. Our findings suggest the need for awareness of the effect of early sexual activity on the development of cervical cancer and its precursor lesions.

A study done in Addis Ababa reported that having more than one sexual partner increases the likelihood of precancerous cervical lesion which is in line with our findings [26]. This finding is supported by several studies done in Nigeria, Swaziland, Tanzania, and Ethiopia [27, 50–52]. A worldwide meta-analysis of forty-one publications reporting on multiple sexual partners and cervical cancer revealed that having multiple sexual partners was significantly associated with the occurrence of cervical cancer [53]. The reason for this could be having sex with multiple partners may increase the chance of transmission of HPV, which is a causative agent for precancerous cervical lesions [54, 55]. The finding of our study underscores the importance of avoiding multiple sexual partners to prevent precancerous cervical lesions.

Our study showed that women having a husband or partner with two or more lifetime sexual partners had higher odds of having a precancerous cervical cancer lesion. Our finding is similar to previous studies done in Ethiopia and Uganda which stated that multiple sexual behaviours of male partners increase the risk of precancerous cervical lesion [26, 38, 56]. Taken together these findings indicate that efforts are needed to raise awareness of the women and their husbands/partners in relation to the risk of multiple sexual partners and precancerous cervical lesion.

The current study did not find an association between HIV and history of STI and precancerous cervical lesion. This is contrary to other studies [20, 51, 57–59]. However, the current study is in line with studies conducted elsewhere in Ethiopia [17, 18, 26]. The reason for the difference might be the small number of women with HIV and history of STI in our study compared to other studies. Because of these conflicting findings, future research with a large sample size in this area might be needed to identify the association between HIV or STI and precancerous cervical lesion.

In our study, contraceptive use was not associated with precancerous cervical lesion and is consistent with previous literature in Ethiopia [18, 26]. Further, a systemic review and meta-analysis done in sub-Saharan Africa indicated that contraceptive use has no relation with the development of precancerous cervical lesion [59]. However, studies have revealed that the use of contraceptive methods increases the risk of precancerous cervical cancer [20, 27]. The possible explanation for the difference could be the later studies were conducted in only urban areas or non-governmental health facilities where the utilization of contraceptive methods was high.

The findings of our study should be interpreted in light of the following limitations. Like other observational studies, association measured in case-control studies may not represent causal effect relationships. Unmatched case-control study design may be influenced by many confounding factors so this may affect the association of outcome and the exposure variable. Further, this study does not show the magnitude of precancerous cervical lesions due to the limitation of the study design. There may be misclassification of cases and controls since the study uses current VIA results to classify the cases and controls. The study was facility-based and may not be representative of the larger group in the population. Moreover, there may be social desirability bias since the study assessed the lifestyle and sexual behaviour of the participants. The strengths of this study include the recruitment of participants from both general and tertiary hospitals; the inclusion of confounders; contraceptive methods use, family history of cervical cancer, and smoking history, which may increase the risks of precancerous cervical lesions.

### The implication of the study

Our study adds to the literature on risk factors for precancerous cervical lesion and highlights the need for further study on this topic in south Ethiopia. The findings have implications for the implementation of public health education that might reduce the development of cervical cancer such as awareness creation on early sexual intercourse, avoiding multiple sexual intercourses, encouraging early screening for cervical cancer. Educating high-risk women should be central to public health response to the precancerous cervical lesion. Therefore, the policymaker needs to establish a clear policy on programmes of education and public information on cervical cancer with the active involvement of health care provider, the community, and the mass media. The present study did not find an association between HIV infection, STI and contraceptive use and precancerous cervical lesion, possible risk factors that require further exploration.

## Conclusion

This study revealed that women age 30–39 years old, monthly income less than or equal to 66USD, age at first sexual intercourse less than or equal to 20 years and a history of having multiple sexual partners were associated with precancerous cervical lesions. Strategies to prevent precancerous cervical lesions should focus on modification of lifestyle and sexual behaviour. The findings of this study highlight several implications for policymakers: targeting older women for cervical cancer screening, addressing inequalities and education relating to risky sexual behaviour may reduce precancerous cervical lesions. A further longitudinal study is needed to identify the awareness of women in relation to cervical cancer screening.

## Supporting information

S1 File Questionnaire. (DOCX)

S2 File STROBE checklist for case-control study. (DOC)

## Abbreviations

CI: Confidence Interval
CSA: Central Statistical Agency
HPV: Human papillomavirus
VIA: Visual Inspection with Acetic Acid
