# Supplementary material for: Risk factors for precancerous cervical lesion among women screened for cervical cancer in south Ethiopia: Unmatched case-control study
Source: PLoS One. 2021 Jul 15;16(7):e0254663. doi: 10.1371/journal.pone.0254663 (PMC8282005; doi:10.1371/journal.pone.0254663)
Supplement: S1 File — (DOCX) [file pone.0254663.s001.docx]

**Consent Form for the Research Project:**

**Risk factors for precancerous cervical lesion among women screened for cervical cancer: A Hospital Based case control study in south Ethiopia.**

I agree to participate in the above research project and give my consent freely.

I understand that the project will be conducted as described in the Information Statement, a copy of which I have retained.

I understand I can withdraw from the project at any time, and do not have to give any reason for withdrawing.

I consent to

- Completing a questionnaire by interview that will take about 10 minutes. The interview is about my socio-demographic characteristics, reproductive history, sexual and behavioral characteristics.
- Allow the researcher accessing my medical record.

I understand that my personal information will remain confidential to the researchers

I have had the opportunity to have questions answered to my satisfaction.

Name of the participants: ___________________ Date: _________ Signature/Finger print:____

Name of data collector_____________________ Date:__________ Signature:____________

English Version Questionnaire

| **Date of data collection** |  |
| --- | --- |
| **Patient code** |  |
| **Health facility name** |  |
| **Case** | **Control** |

**Part I- Socio Demographic characteristics**

| **SN** | **Questions and Filters** | **Response & Coding Categories** | **Skip** |
| --- | --- | --- | --- |
| 101 | How old are you? (Complete in years) | _­­­­­­­­­­________ Years |  |
| 102 | Where is your permanent residence? | 1. Urban 2. Rural |  |
| 103 | What is your religion? | 1. Orthodox 2. Muslim 3. Protestant 4. Catholic 5. Other, Specify ____________ |  |
| 104 | What is your marital status? | 1. Married 2. Single 3. Widowed 4. Divorced/separated |  |
| 105 | What is your current educational status? | 1. Cannot read and write  2. Able to read and write but with no schooling  3. Primary school – 1- 4 grade  4. Primary school – 5- 8 grade  5. Secondary school  6. College diploma and above |  |
| 106 | What is your current occupational status? | 1. Government employee 2. Non-governmental employee 3. Merchant 4. Farmer 5. Daily laborer 6. Student 7. Housewife (unemployed) 8. Other specify **___________** |  |
| 107 | What is your husband educational status currently? | 1. Cannot read and write  2. Able to read and write but with no schooling  3. Primary school – 1- 4 grade  4. Primary school – 5- 8 grade  5. Secondary school  6. College diploma and above |  |
| 108 | What is your monthly total household income? | ___________ETB |  |
| 109 | Body Mass Index | Height(cm)___________  Weight(kg)___________ |  |

**Part II- Reproductive History of Participants**

| **SN** | **Questions and Filters** | **Response & Coding Categories** | **Skip** |
| --- | --- | --- | --- |
| 201 | How many pregnancies have you had so far? | _____________ | If None skip to 205 |
| 202 | How many living children do you have? | _______________ |  |
| 203 | History of Recurrent abortion | 1. Yes 2. No |  |
| 204 | Age at first pregnancy | _­­­­­­­­­­________ Years |  |
| 205 | Age at Menarche | _­­­­­­­­­­________ Years |  |
| 206 | How do you describe your menstrual pattern? | 1. Regular 2. Sometimes Irregular 3. Always Irregular 4. No menses |  |
| 207 | Are you currently using hormonal contraceptive? | 1. Yes 2. No | If No skip to 209 |
| 208 | Type of contraceptive you are using? | 1. Oral contraceptive pills 2. Injectable Contraceptive 3. Implants 4. IUCD 5. Other, specify ___________ |  |
| 209 | Have you ever used Oral Contraceptive Pills? | 1. Yes 2. No |  |
| 210 | Interval between births? | --------------------- years |  |

**Part III Lifestyle and sexual behavior**

| **SN** | **Questions and Filters** | **Response & Coding Categories** | **Skip** |
| --- | --- | --- | --- |
| 301 | Age at first sexual intercourse | _­­­­­­­­­­________ Years |  |
| 302 | Life time number of sexual partners | 1. None 2. One 3. Two 4. Three or more |  |
| 303 | Number of sexual partners of your partner | 1. None 2. One 3. Two 4. Three or more |  |
| 304 | History of sexually transmitted disease | 1. Yes 2. No |  |
| 305 | Partner history of sexually transmitted disease? | 1. Yes 2. No |  |
| 306 | Have you ever had post coital bleeding | 1. Yes 2. No |  |
| 307 | Do you have a family history of cervical cancer? | 1. Yes 2. No |  |
| 308 | Have you been screened for cervical cancer before? | 1. Yes 2. No |  |
| 309 | HIV status | 1. Reactive 2. Non-reactive |  |
| 310 | Smoking exposure | 1. Yes 2. No |  |
| 311 | Any form of chronic illness currently being treated for? | 1. Yes 2. No |  |
| 312 | Vulval washing after every sexual intercourse | 1. Yes 2. No |  |
